# Transplanted embryonic retinal stem cells have the potential to repair the injured retina in mice

**DOI:** 10.1186/s12886-020-01795-1

**Published:** 2021-01-09

**Authors:** Xia Feng, Peng Chen, Xin Zhao, Jing Wang, Hong Wang

**Affiliations:** 1 Department of Ophthalmology, Shandong Provincial Western Hospital, Shandong Provincial ENT Hospital, Jinan, China; 2 Department of Ophthalmology, Shandong Provincial Hospital Affiliated to Shandong First Medical University, No324, Jingwu Road, 250021 Jinan, China

**Keywords:** Retinal stem cell, Retinal injury mice, Transplantation, Intravitreal injection

## Abstract

**Background:**

Stem cell transplantation has been reported as one of the promising strategies to treat retinal degenerative diseases. But, the application and the role of retina stem cells (RSCs) in the treatment of patients with retinal degenerative diseases have not been fully revealed. This study aimed to investigate the potential role of transplantation of the embryo-derived RSCs into the vitreous cavity in repairing the damaged retina in mice.

**Methods:**

RSCs were isolated from Kunming mice E17 embryonic retina and ciliary body tissues, and labeled with 5-bromo-2’-deoxyuridin (BrdU). Retinal optic nerve crush injury was induced in left eyes in male Kunming mice by ring clamping the optic nerve. The 6th -generation of BrdU-labeled RSCs were transplanted into the damaged retina by the intravitreal injection, and saline injected eyes were used as the control. Hematoxylin and eosin histological staining, and BrdU, Nestin and Pax6 immunostaining were performed. Electroretinogram (ERG) was used for assessing the electrical activity of the retina.

**Results:**

Embryo-derived RSCs were identified by the positive stains of Pax6 and Nestin. BrdU incorporation was detected in the majority of RSCs. The damaged retina showed cellular nuclear disintegration and fragmentation in the retinal tissue which progressed over the periods of clamping time, and decreased amplitudes of a and b waves in ERG. In the damaged retina with RSCs transplantation, the positive staining for BrdU, Pax6 and Nestin were revealed on the retinal surface. Notably, RSCs migrated into the retinal ganglion cell layer and inner nuclear. Transplanted RSCs significantly elevated the amplitudes of a waves in retina injured eyes.

**Conclusions:**

Embryonic RSCs have similar characteristics to neural stem cells. Transplantation of RSCs by intravitreal injection would be able to repair the damaged retina.

## Background

Blindness eye diseases traditionally include cataract, corneal infection, and refractive stromal lesions. With the development of the basic research and clinical technologies, many of the oculopathy are becoming treatable. However, the spectrum for blindness eye disease is changing remarkably. Currently, retinal disease is the leading cause of vision loss worldwidely [1]. Age-related macular degeneration (AMD) and retinitis pigmentosa (RP) are 2 representative retinal diseases [1]. Photoreceptors (PRs) is responsible for conversion of the light into electrical signals for further process and integration. Anatomically, PRs are in contact with the retinal pigment epithelium (RPE). RPE involves the transport of nutrients, the recycling of proteins, and the elimination of photoreceptor debris. RPE also secretes some growth factors [2]. Therefore, dysfunctions or death of RPE cells can induce loss of PRs, leading to damage of vision and in some cases ultimately causing blindness. Of note, RPE are not able to endogenously regenerate [3]. Stem cells have the key ability to self-renew and differentiate to various types of cells. It has been shown that stem cells are becoming an attractive source of cell therapy in replacing or repairing damaged RPE and PRs [4]. Retinal stem cell therapy is one of the promising therapeutic alternatives to recover vision in patients with retinal disease [2, 5].

Three classes of stem or progenitor cells are utilized for cell therapy, including pluripotent stem cells (PSCs), fetal cells, and postnatal/adult cells. Cell-based therapies for retinal diseases that are currently under investigation usually use PSCs either embryonic stem cells (ESCs) or induced pluripotent stem cells (iPSCs) [3, 4, 6–9]. To date, a lot of studies have shown that stem cells or stem-cell-derived cells improve the survival and function of host cells, not by producing the missing cells, but by secreting growth factors [10]. The transplanted cells can migrate and integrate into the various layers of the retina, potentially induced by neural differentiation stimulating factors [11–14]. iPSCs are similar to ESCs, but derived by de-differentiating fully differentiated adult somatic cells (such as skin fibroblasts or white blood cells) into stem cells and then re-differentiating them into target cells [4]. Evidently, there are theoretical ethical advantages in using iPSCs for transplantation; but potential risks such as development of malignancy are present due to unknown mechanism of transformation and transcription factors [15, 16]. Therapeutic RSCs can be delivered to the intravitreal space, posing a less risk than subretinal injection since the latter can result in retinal detachment [6]. As such, RSCs derived from ESCs appeared to have more benefits in the treatment of retinal disease. However, the application and the role of RSCs in the treatment of retinal degenerative diseases have not been fully revealed. At present, many issues in the application of RSCs need be explored and solved, such as the source of RSCs used for transplantation, transplantation methods, local survival periods and electrophysiological function maintenance, etc.

This study aimed to discuss the current stem cell transplantation approaches, and to investigate the feasibility and significance of embryonic RSCs transplantation by intravitreal delivery in a retinal damaged mice model. We found that embryo-derived RSCs have similar characteristics to neural stem cells (NSCs), which were evidenced by the presence of Pax6 and Nestin expressions. Transplanted RSCs were observed in all layers of the retina, suggesting that they have potential to function to repair the damaged retina. Accordingly, these findings herein provided theoretic evidence that facilitate the studies of RSCs transplantation in the treatment of retinal disease.

## Methods

### Animals

Healthy Kunming mice aged 5–6 weeks (*n* = 15; 3 pregnant and 12 non-pregnant) were purchased from Beijing HuaFuKang Biotechnology Co., LTD (Beijing, China), and maintained on a regular diurnal lighting cycle (12:12 light:dark) with ad libitum access to food and water under specific-pathogen-free conditions at the Central Animal Care Services of Shandong University. Carbon dioxide inhalation was used for euthanasia. Mice were placed into a chamber initially containing room air with the lid closed, and then 100% compressed carbon dioxide was infused for 30 s at a displacement rate of 30% of the chamber volume per minute to induce a rapid anesthesia, followed by an increasing flow rate and continued exposure of 3 min for death induction. Upon completion of the procedure, death was confirmed by absence of all muscle activity and vital signs (no breath, mydriasis) for at least 30 s for each animal. This study was approved by the Shandong Provincial ENT Hospital (Shandong Provincial Western Hospital) Ethical Committee (Project No: XYK-20,200,701), and all procedures were conducted in accordance with the National Institutes of Health Guide for the Care and Use of Laboratory Animals.

### Preparation of retinal stem cells

**Fig. 1:**
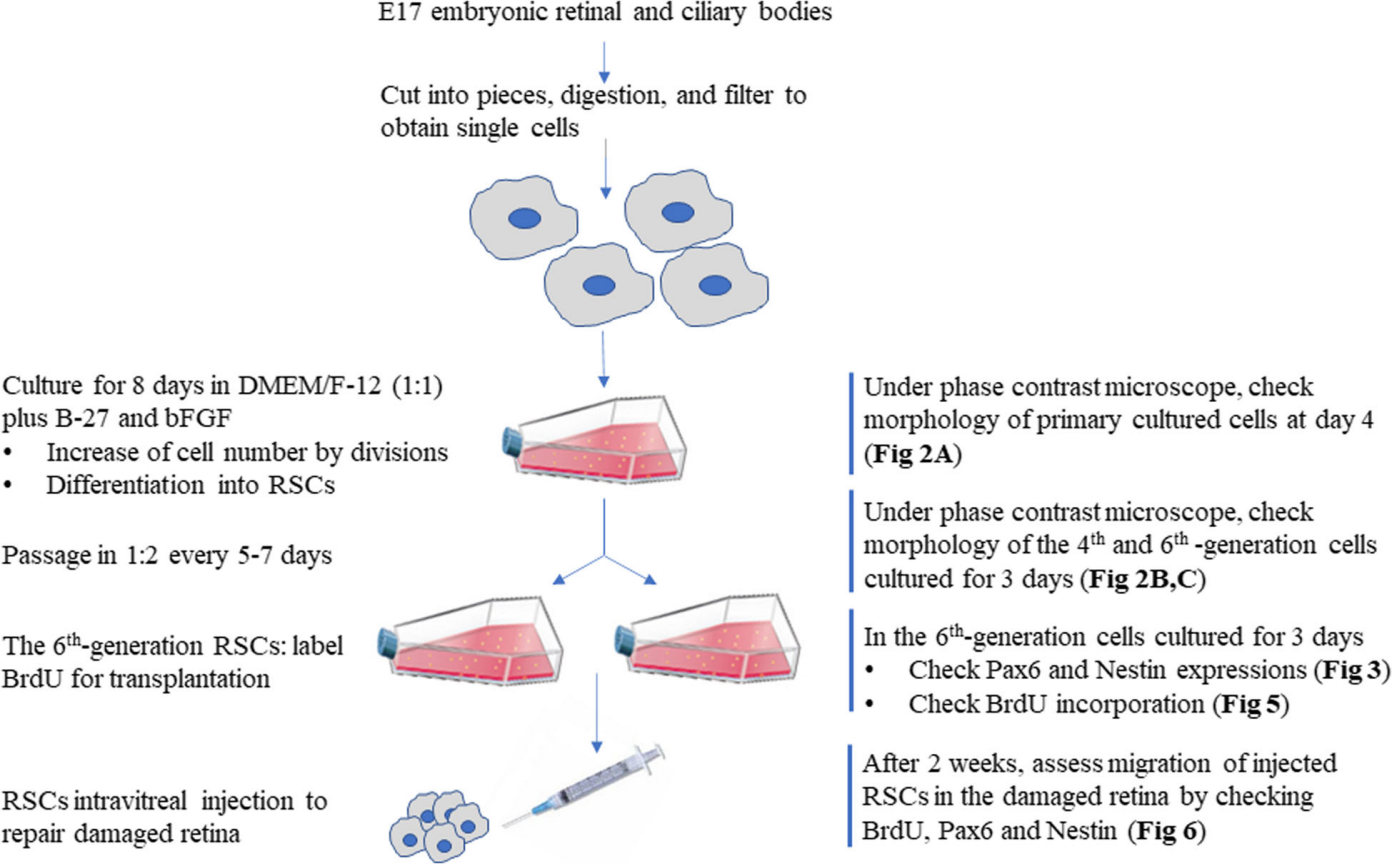
Schematic of the preparation of retinal stem cell

At the gestational age of 17 days, pregnant Kunming mice (*n* = 3) were euthanized, and embryos were immediately excised. As described previously [17–19], both the retina and ciliary body tissue including the pigmented layer-the ciliary marginal zone (CMZ) were cut into small pieces under a dissecting microscope for RSCs isolation (Fig. 1). The resulting tissues were digested using 100 U/mL collagenase (cat. no. C0130, Sigma-Aldrich, Shanghai, China) for 1 h, followed by 0.25% trypsin (cat. no. 15,050,057, Invitrogen, Shanghai, China) digestion for 15 min. Single cell was obtained by passing the suspension through use of a stainless-steel filter with 50 µm porous. Subsequently, the cells were transferred to a 25 mL culture flask at an inoculum density of 5 × 10^4^ /mL in a 1:1 nutrient mixture of Dulbecco’s modified Eagle’s medium (DMEM) and F-12 (cat. no. 11,330,057, Gibco, Gaithersburg, MD, USA), supplemented with 1% B-27 (cat. no. 17,504,044, Gibco) and 20 ng/ml bFGF (cat. no. PHG0368, Gibco). Cells were then cultured at 37 °C under 5% CO_2_. After 8 days of the culture, the primary suspensions of the cellular clusters were passaged in a 1:2 ratio following 1 h collagenase digestion and disruption as a result of being forced through a sterile syringe for 5 times. The process was repeated every 5–7 days. The sixth-generation cells were identified for RSCs using indirect immunofluorescence staining for Nestin and Pax6.

### 5-Bromo-2’-deoxyuridine (BrdU) labeling of RSCs

RSCs from the sixth-generation cultures were added to Poly-L-lysine packaged sterilized culture dishes and cultured for 3 days. Thereafter, 200 µg/ml of BrdU solution (cat no. 19–160, Sigma-Aldrich) was added to the cells. The cells were cultured for 2 days in the presence of BrdU. Incorporation of BrdU was assessed using immunohistochemical staining.

### Optic nerve crush injury

Twelve male Kunming mice aged 8 months were anesthetized with 2% pentobarbital sodium by intraperitoneal injection. This allowed us to drop tetracaine solution (cat. no. 4512, Sigma Pharmaceuticals, North Liberty, IA, USA) into the left eyes for surface anesthesia, and clamp optic nerve with a micro-artery clamp for 15 s at 2 mm behind the globe. The right eyes without any treatment were used as the controls. Retinal slicing and hematoxylin and eosin (H&E) histological staining was performed 1 week after procedure.

### Retinal stem cell transplantation

The standard intravitreal injection for RSCs transplantation was used as described previously [20]. Twelve Kunming male mice with left injured retina were randomly divided into RSCs injection group (*n* = 6) and PBS injection group (*n* = 6) in order to investigate the role of transplanted RSCs. Mice were anesthetized with 2% pentobarbital sodium by intraperitoneal injection, and the limbs and head were fixed well to allow access to the left (operative) eyes. Under a surgical microscope, a 10 µl micro-syringe connected to a 30G needle was inserted into the vitreous cavity from the corneoscleral limbus of the left injured eye in 3 mice, and 2 µl cell suspension of RSCs (3 × 10^4^ cells/µl) from the 6th -generation cultures was carefully injected into the vitreous cavity. All injections were successful, which was verified by without bleeding after observing for 30 s. The left injured retina in the other 6 mice were injected with 2 µl of sterile PBS in the same manner, serving as the controls. After 2 weeks, mice were thereafter euthanized, and eyeballs were removed for immunohistochemical staining. Of note, the operator was unaware of the group allocation and disclosed it until results were obtained and analyzed.

### Electroretinogram (ERG)

Electroretinogram (ERG) was performed to assess the electrical activity of the retina. The German RETLport visual physiological detection system was used for ERG detection. The recording electrode was a self-made acupuncture needle electrode, placed on the limbus, and the reference electrode and ground electrode were placed under the skin of the cheek and tail, respectively. Before the test, the mouse was placed in a dark room for half an hour to adapt to the dark. After the dark adaptation, the pentobarbital sodium was anesthetized intraperitoneally, the cheeks and tail hair were shaved, and the experiment was performed in an electrically shielded dark room. The passband was set at 5–30 Hz, the impedance between the recording electrode and the reference electrode was < 5KΩ; the sampling time was 250 ms, and the waveform was superimposed and averaged for 200 times.

### H&E staining

H&E staining was performed on the sections of paraffin-embedded tissue according to the standard methods.

### Immunohistochemistry staining

The sections of paraffin-embedded tissue were initially placed into a temperature-controlled chamber at 60 °C for 30 min, followed by 10 min-deparaffinization in xylene and a series of gradient ethanol solutions: 100% ethanol for 1 min, 95% for 1 min, and 70% for 1 min. After hydration in distilled water for 2 min, the slices were immersed into 3% hydrogen peroxide for 15 min at room temperature to block endogenous peroxidase activity. For antigen retrieval, the slices were immersed into 2 mol/L HCl for 1 h and then dried at room temperature, followed by 0.1 mol/L NaOH for 2 s and rinse with PBS for 2 min. This step was repeated 3 times.

Subsequently, sections were stained with mouse anti-BrdU antibody (1:100, cat. no. ab8152, Abcam, Cambridge, United Kingdom) overnight at 4 °C. After rinsed 3 times with PBS, sections were incubated with sheep anti-mouse IgG solution (1:50, cat. no. ab6710, Abcam) at room temperature for 30 min. 3,3 diaminobenzidine (DAB) coloration was performed under microscope, and hematoxylin was used to stain the nuclei for 3 min. The specimens were then rinsed with water, dehydrated, and sealed for observation and imaging. Representative images were taken with optical microscope (Leica DM2500, Will and International Trade Co., LTD, Hong Kong).

### Indirect immunofluorescence staining

Indirect immunofluorescence staining of Nestin and Pax6 were performed on cold acetone-fixed frozen tissue sections. After 3 washes with PBS, the sections were blocked in 10% goat serum for 1 h to decrease background signal. Rabbit anti-Pax6 (1:100, cat. no. ab195045, Abcam) and mouse anti-Nestin (1:100, cat. no. ab6320, Abcam) were added and incubated overnight at 4 ℃ in a wet box. After 5 washes with PBS, Alex488 and 594 conjugated goat anti-rabbit or mouse IgG (1:2,000, cat. no. A-11,034, cat. no. A-11,032, Invitrogen) were applied at 37^o^C for 1 h in the dark room. Notably, the staining when the primary antibodies were omitted and only the secondary antibodies were applied were used as the blank controls. After 5 washes with PBS, DAPI was added and incubated in dark for 2 min. The specimens were then sealed with glycerin and immediately observed and photographed under a fluorescence microscope (BD-YG500, Shenzhen Boshida Optical Instrument Co., Ltd., Shenzhen, China).

### Statistical analysis

Data are shown as mean ± standard deviation. Two tailed *t* test was performed through use of Graphpad Prism 8.2.1 software (GraphPad Software, Inc., San Diego, CA, USA). *P < 0.05* is considered as having significant differences.

## Results

### Culture and identification of retinal stem cells

**Fig. 2:**
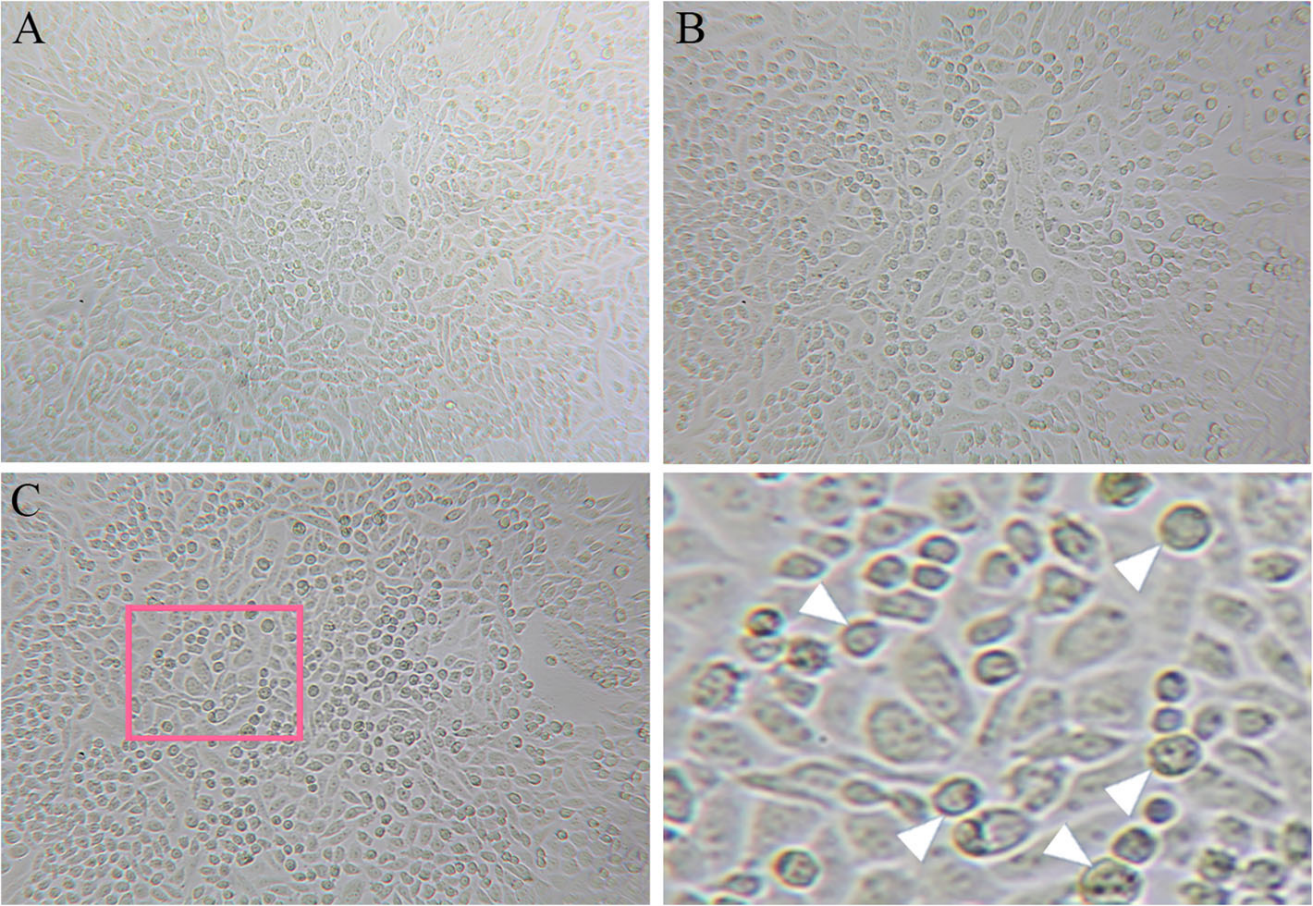
Generation of mouse retinal stem cells. Retinal stem cells were isolated from embryonic E17 retinal and ciliary bodies. **a** Phase contrast imaging of primary cultured cells at 4 days. Most of the cells showed fusiform or round phenotype. **b** Three days after extending to the 4th generation, cell densities increased, and the majority of cells were round. **c** After extending to the 6th generation, the cells grew well, and round cell density increased as indicated by the arrowhead. *Magnification: ×200 for all images. A high-power view of the selected area was presented on the right*

Mouse retinal and ciliary bodies from E17 embryos were used to isolate RSCs. Under phase contrast microscope, most of the primary cultured cells showed fusiform or round phenotype, and a few of cells were long and thin strip cells at day 4 after culture (Fig. 2a). The cell vitality was 40–50%. For the 4th -generation cells, 3 days after culture, the cell density was increased, and most of the cells were round (Fig. 2b). After extending to the sixth generation, the cells grew well, and round cells increased obviously (Fig. 2c). This indicated that the cultured cells possessed the morphological characteristics of RSCs.

**Fig. 3:**
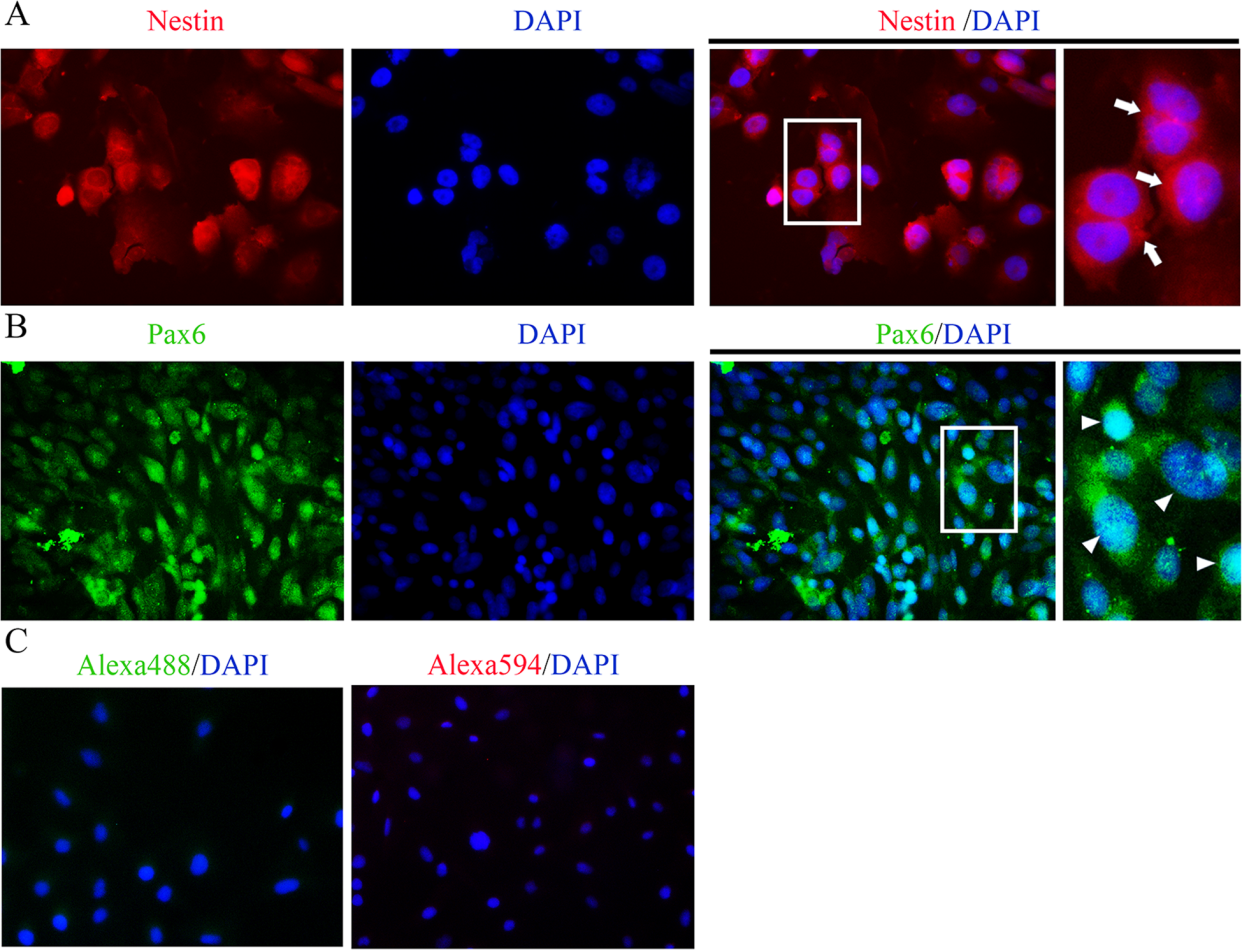
Immunofluorescence staining of Pax6 and Nestin on retinal stem cells. Indirect immunofluorescence confocal microscopy was used to image Pax6 and Nestin on the sixth-passage retinal stem cells cultured for 3 days. Nuclei were labeled with DAPI. **a** Nestin was predominantly localized in cytoplasm (arrow). **b** Pax6 was observed mainly in nuclei (arrowhead). **c** Primary antibodies were omitted and only the secondary antibodies Alexa488 or 594 conjugated goat anti-rabbit or mouse IgG were applied for the blank controls. *Magnification: x200 for Pax6, x640 for Nestin*. *A high-power view of the selected area was presented on the right*

The sixth-generation cells were cultured, and after 3 days, Pax6 and Nestin were stained using indirect immunofluorescence staining for verification of RSCs. It has been demonstrated that Pax6 is required for the multipotent state of retinal progenitor cells [21]. Nestin, a cytoskeletal intermediate filament, is initially characterized in neural stem cells. However, current extensive evidence suggested that Nestin plays an essential role in stem cell functions, including self-renewal, differentiation and migration [22]. Therefore, Pax6 and Nestin are commonly used as the markers of retinal stem cells. Our results showed that Nestin was predominantly localized in cytoplasm, and Pax6 was mainly observed in nuclei (Fig. 3), suggesting that the 6th -generation cells were RSCs.

### Alterations of retinal histology and ERG in the mice with optic nerve crush injury

**Fig. 4:**
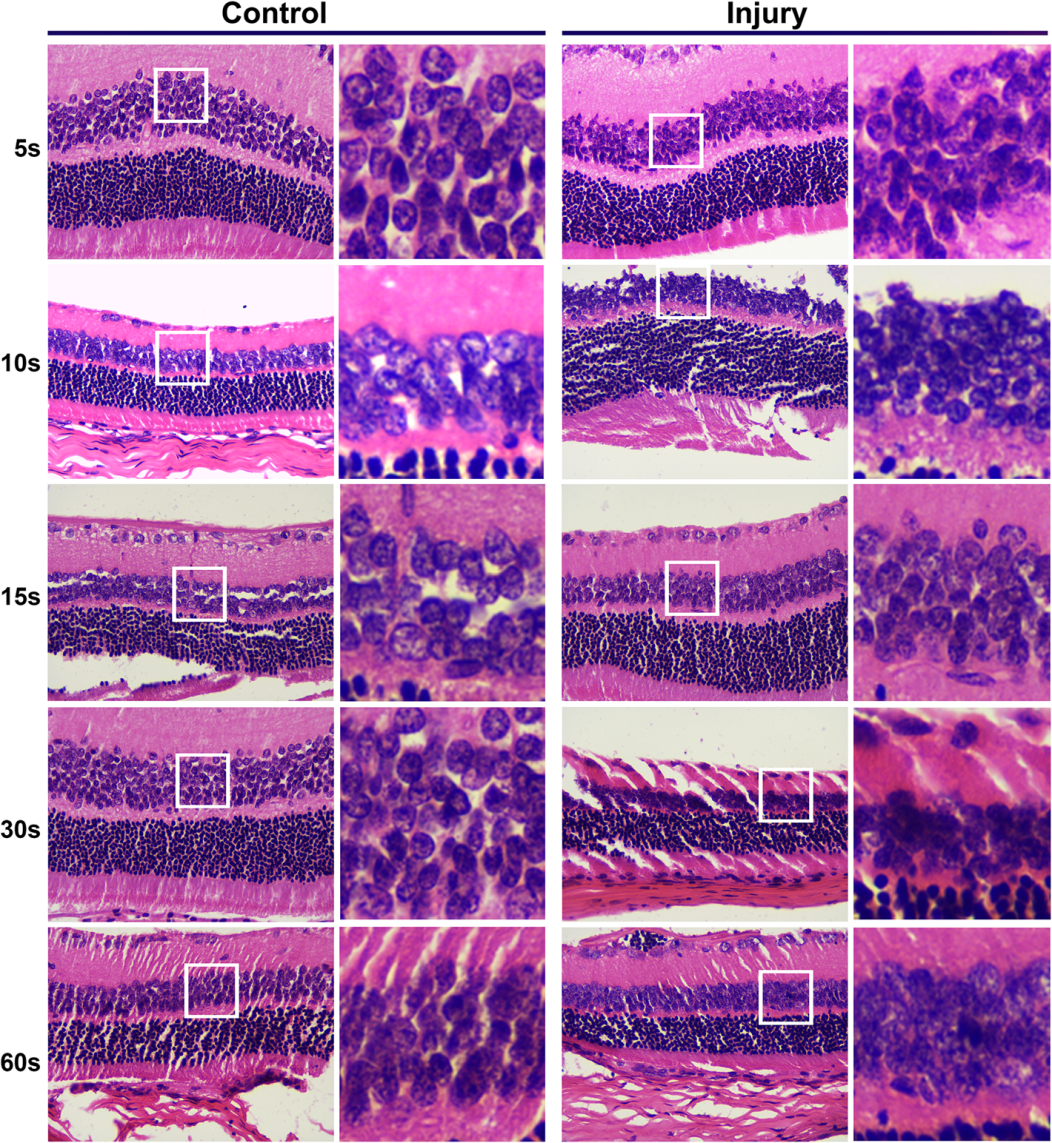
Histological staining of retinal morphology in optic nerve crush injury model. Optic nerve crush injury model was performed with the indicated time periods for clamping the optic nerve. Retinal structure was revealed by use of Hematoxylin and Eosin (H&E) staining 1 week after injury. Images captured at high magnifications with a microscope showed that cellular nuclear disintegration and fragmentation in the retinal tissue of the injured group, and the damage progressed over the periods of clamping time. The retinal cell nuclei of the control group were well arranged with normal morphology. Representative images from one of the retinal injured mice were provided. *Magnification: x400*. *A high-power view of the selected area was presented on the right*

Optic nerve crush injury model was induced by clamping optic nerve through use of a micro-artery clamp. Clamping decreased and/or blocked the blood supply of the central retinal artery running in the optic nerve, leading to retinal ischemia, cellular apoptosis and tissue necrosis. One week after the operation, retinal structure was checked using H&E staining. Images captured at high magnifications with a microscope showed that the retinal cell nuclei of the control group were well arranged with normal morphologies. Nevertheless, cellular nuclear disintegration and fragmentation were observed in the retinal tissue of the injured group. Of note, the morphological changes of the retinal granular layer in the injured group progressed over the periods of clamping time (in seconds). At 15 s following clamping, obvious pathological changes were found and characterized by the disordered granular layer cell structure, the ruptured cell membranes, and the collapsed cytoplasm and nuclei, especially in the outer granular layer. At 60 s, the cellular morphologies were dramatically damaged (Fig. 4). As the degree of cell damage at 15 s was closer to that seen in the patients with retinal diseases, we chose 15 s for optic nerve clamping time to establish the retinal damage model.

In addition, the data from ERG showed that the amplitudes of a wave, particularly the b wave were significantly lower in the injury group than that in the control group (Table 1). This indicates that both the outer and the inner nuclear layers was damaged by the clamping procedure.

**Table 1:** Comparison of the amplitudes of the ERG between the injury and the control groups

	Injury (*n* = 6)	CTL (*n* = 6)	*P value*
a wave (µV)	-38.74 ± 5.28	-64.42 ± 5.29	*0.000*
b wave (µV)	87.72 ± 10.86	168.4 ± 13.92	*0.000*

### Retinal stem cells transplantation for repair of the damaged retina

**Fig. 5:**
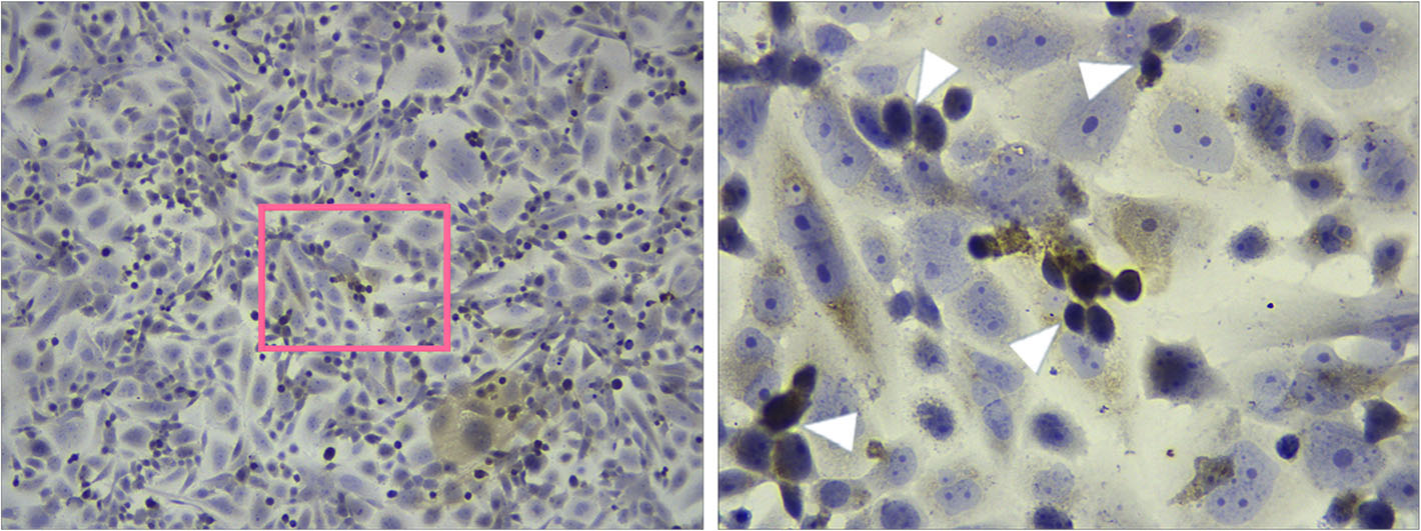
Immunohistochemical staining of BrdU on retinal stem cells. The sixth-generation retinal stem cells were cultured for 3 days after passaging. Immunohistochemical staining of BrdU were then performed on fixed cells. BrdU was incorporated into most of retinal stem cells, evidenced by yellow-brown nuclei (arrowhead). *Magnification: ×200. A high-power view of the selected area was presented on the right*

The 6th -passage RSCs labeled with BrdU were used for transplantation. Since it can be incorporated into the newly synthesized DNA of replicating cells, BrdU is frequently used in analysis of neural stem cell biology, in particular to label and to fate-map dividing cells [23]. Firstly, incorporation of BrdU was assessed on the fixed cells. Immunohistochemical staining show that approximately 90% of RSCs were labeled with BrdU, evidenced by brownish staining in nuclei (Fig. 5).

**Fig. 6:**
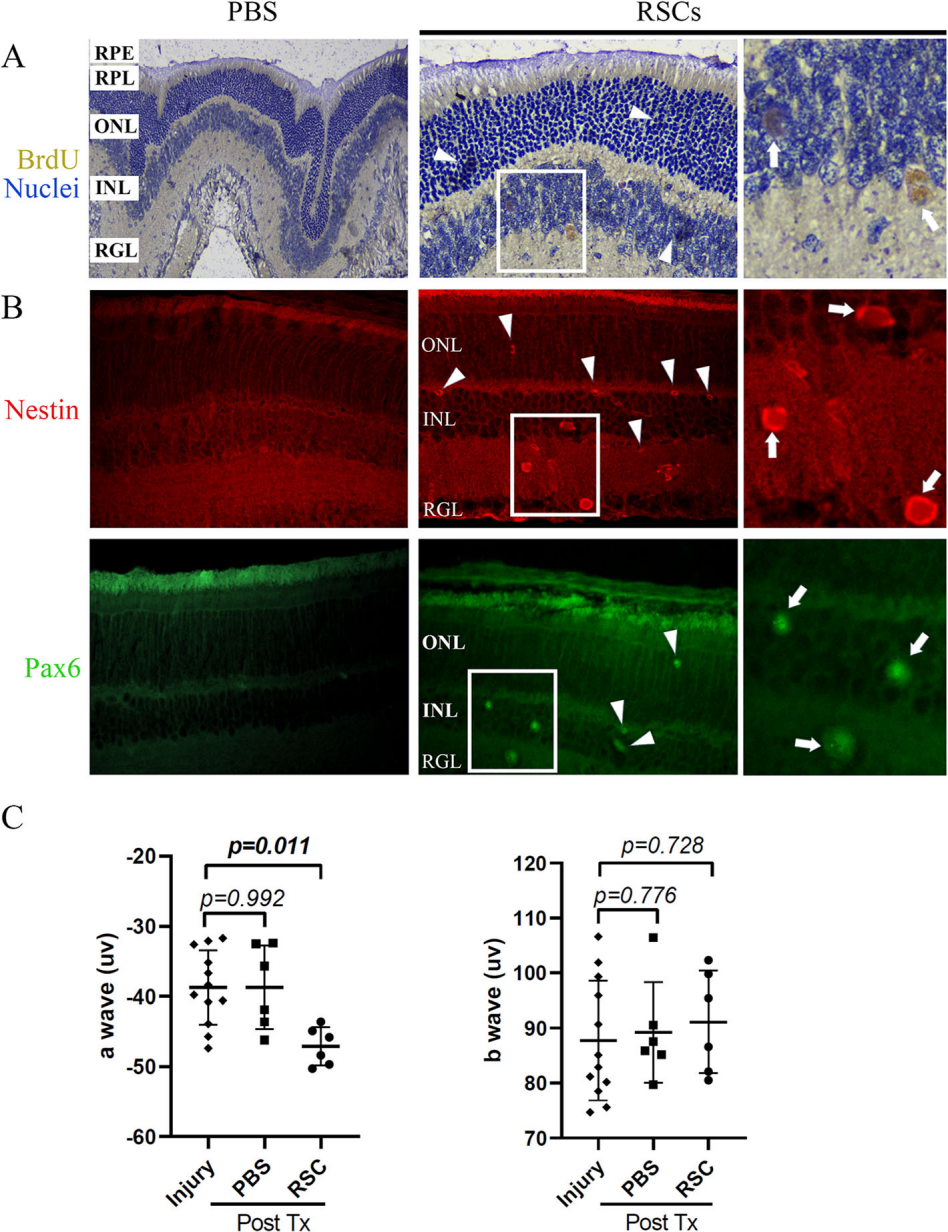
Transplanted retinal stem cells were detected in optic nerve crush injury model. The sixth-passage retinal stem cells (RSCs) labeled with BrdU were transplanted to the injured eyes for 2 weeks as described in the Methods part. PBS injected eyes were used as the controls. **a** Localization of BrdU-retaining cells by immunohistochemistry staining. **b** Immunofluorescence staining of Nestin and Pax6. In retinal injured eyes transplanted with RSCs, nuclear staining of BrdU and Pax6, and cytoplasmic staining of Nestin, were revealed on the surface of retina, predominantly between the RGL and INL (arrow). A few of positive stains for BrdU, Pax6, and Nestin were also detected in the ONL (arrowhead). In the PBS injected eyes, all layer of the retina remained unstained. Representative images from one of the retinal injured mice with RSCs or PBS injection were provided. *Magnification: x400 for all images*. *A high-power view of the selected area was presented on the right. RGL: retinal ganglion layer (ganglion cell), INL: inner nuclear layer (bipolar cell, Müller glia, and horizontal cell), ONL: outer nuclear layer (Rod and cone cell), RPL: retinal photoreceptor layer, RPE: retinal pigment epithelium.***c** Electroretinogram (ERG) was performed pre and post RSCs or PBS transplantation (Tx) in the retinal damaged mice. Compared to PBS transplantations, RSCs transplantation significantly elevated the amplitudes of a waves. *n* = 6. Two tailed *t* test. Data are shown as mean ± standard deviation. The *p* values are shown in the figures

RSC suspensions were then transplanted into the damaged retina using the intravitreal injection method. Two weeks post-operation, pathological sections were stained for BrdU, Pax6 and Nestin. In the transplanted retina with RSCs, nuclear staining of BrdU and Pax6, and cytoplasmic staining of Nestin, were revealed on the surface of retina, predominantly between the retinal ganglion layer and inner nuclear layer (Fig. 6a, b). Notably, a few of positive stains were also detected in the outer nuclear layer in the damaged eyes that accepted transplantation of RSCs (Fig. 6a, b). In the control eyes, all layers of the retina were not positively stained. Representative images from one of the retinal injured mice with RSCs or PBS injections were provided.

To assess if the transplanted RSCs have the ability to repair the electrical activity of the damaged retina, we performed ERG experiments in mice with optic nerve crush injury receiving either RSCs or PBS transplant. Compared to the PBS transplantations, RSCs significantly augmented the amplitudes of a wave (Fig. 6c). The b waves did not show remarkable differences between RSCs and PBS transplanted retina. This indicated that after RSCs transplantation, the function of the outer layer of the injured retina recovered at least to a certain extent. Nevertheless, transplanted RSCs did not improve the function of the inner layer of the retina, possibly associated with the differentiation of RSCs and the limited time we had to observe.

## Discussion

Stem cell engineering has opened a new avenue for repairing damaged nervous tissues. Following transplantation of stem cells into eyes, they further integrate into the retinal microenvironment, and then proliferate and differentiate into target cells, to regenerate the damaged neurons [6]. This offers recovery and reconstruction of the retinal function, with opportunities to treat irreversible blindness ophthalmopathy.

Previous work has demonstrated that the injection of ESCs into the subretinal space of rats effectively alleviated photoreceptor cell degeneration and death [24]. NSCs have been found in the embryonic nervous system and in certain parts of the adult brain. Due to continuous self-renewal and proliferative ability, these cells can differentiate into specific neurons and glial cells. Recently, it has been reported that NSCs were successfully integrated into the various layers of the retina [25–27]. The major challenge is how the NSCs differentiate into mature retinal cells. Some studies have shown that differentiation was associated with the growth environment of the cell [28–30]. In addition, there is an ongoing clinical trial from ReNeuron about the application of the human retinal progenitor cell therapy in the treatment of patients with retinitis pigmentosa (NCT02464436). In the present study, we isolated, cultured, and propagated mouse RSCs from E17 embryos. After extending to the 4th generation, the cells presented with the phenotype of RSCs. To further verify if these cells were stem cells and had the characteristics of NSCs, we stained the stem cell marker Pax6 and NSC-specific marker Nestin [21, 22]. Cultured RSCs not only had stem cell morphologies under phase contrast microscope, but also highly expressed Pax6 and Nestin, indicating that the cultured RSCs belonged to the NSC family. Therefore, embryonic RSCs might be becoming prospective cells for retinal transplantation.

Considering the RSC as the most suitable seed cell, we further examined the feasibility of RSCs transplantation in the treatment of the damaged retina. In general, there are currently 2 types of transplantation methods: subretinal space injection and vitreous cavity injection [6, 31]. Maintaining intraocular structure and preventing immune rejection is crucial for the survival of transplanted cells. In the case of subretinal space injection, it is often difficult to avoid blood retinal barrier disruption within the eyeball, which can cause severe swelling as well as degeneration and/or death of transplanted cells [31]. The intravitreal injection is a simplest feasible way in clinical practice for intraocular medication, which allows the eyeball to remain intact and reduces the occurrence of damage to the retina barrier. Moreover, this method offers a clear field of view during operation [6, 31, 32]. Although both of these grafting methods are capable of integrating seed cells into the retina, some studies recently compared the 2 methods, showing that trauma induced by the subretinal injection is considerably greater leading to retinal detachment [17]. Therefore, the intravitreal injection method is commonly preferred for delivery of medication or stem cell. In our study, we delivered BrdU-labeled RSCs into vitreous cavity for cell transplantation. Upon examination of the retina post-operation till 2 weeks no infections or bleeding were observed. Close observation of sectioned specimens revealed a complete intraocular structure with apparent anatomic arrangement. These findings suggest that intravitreal injection is a safe delivery way with less trauma for transplantation.

In order to gain an insight into the cell arrangement, we investigated the effect of transplanted RSCs on the retinal neuron composition. Retinal neurons are typically divided into 3 layers: ganglion cells, bipolar cells, and photoreceptor cells, from the inner to outermost layer, respectively [33]. Photoreceptor cells convert light stimuli into nerve impulses, the bipolar cells transfer nerve impulses to ganglion cells, and the nerve impulses transfer through the ganglion cells nerve fiber to the optical center, which produces the visual [33]. As the retinal ganglion cells are associated to nerve fibers, any mechanical damage to the optic nerve can block axoplasmic transport of the ganglion cells, leading to direct impairment of ganglion cell nutrition [33]. The pathology of such retinal damage and visual function lesions are mimicked by ring clamping of the optic nerve [34].

Here, we firstly performed optic nerve crush injury model by ring clamping the optic nerve. One week after operation, images captured at high magnifications with a microscope showed nuclear disintegration and fragmentation in the retinal tissue of the injured group, and the damage progressed over the periods of clamping time. Retinal ganglion cells and other inner retinal cells are mainly affected by ischemic injury since the central retinal artery mainly supply inner two thirds of retina. Consistently, the findings from ERG showed that the amplitudes of a wave, in particular the b wave, was significantly decreased in the injured retina compared to the control. Therefore, more dramatic injury of the inner nuclear layers was disclosed following optic nerve crush. We then attempted to transplant the BrdU-labeled RSCs in close proximity to the ganglion cell layer, which allowed us to assess the effect of the damaged optic nerves directly. Notably, the percentage of the BrdU-positive RSCs was greater than 90%, indicating that almost all the RSCs were labeled. Characterization of transplanted tissues was thereafter carried out after 2 weeks. Our results showed that the BrdU-positive cells were present in vitreous cavity, the retinal surface and the ganglion cell layer, indicating a successful transplantation of the RSCs and their entry into the damaged retina. This is further demonstrated by the presence of Pax6 and Nestin-positive cells in the retina, predominantly between the retinal ganglion layer and inner nuclear layer. Morphological evaluation also suggested that the transplanted RSCs were integrated into the host retina, implying its potential to substitute for the damaged cells. This observation was consistent with the previous reports [35, 36]. The preliminary data from ERG further demonstrated the potential repair ability of RSCs, which was restricted in the outer layer of the damaged retina. It may be associated with the differentiation of RSCs and the limited time we had to observe. Additionally, a large number of RSCs were injected into the vitreous cavity, and only a few cells were observed to survive in the vitreous and retina. It was speculated that it might be related to cellular apoptosis induced by autoimmune response. How to keep and increase RSCs survival in living tissues is 1 of the major challenges in the research of RSCs transplantation. Whether RSCs can smoothly migrate or differentiate into inner retinal cells remains to be further studied. Notably, the observation time of the current study was relatively short, and the key synaptic link may not have been developed yet.

Several studies have shown that the transplanted NSCs in retinas can partially differentiate into neurons [37–39], but generally depend on microenvironment of the host [40, 41]. In addition, it has been reported that this differentiation was also related to the host age. Li N et al. showed that the integration ability of stem cells transplanted into the vitreous cavity of rats decreased with the increase of host age [42]. Nevertheless, this phenomenon may be associated with a range of other factors such as the existence of a large number of undifferentiated cells in the neonatal host, an imperfect barrier function, and some growth factors promoting cell migration [43]. Furthermore, it has been shown that when the graft contains more mature cells it easily forms aggregates of cells or rosettes, which disrupt the integration of graft and host and thus prevent the transplanted cells from reconstructing the retinal function [44]. However, embryonic cells or stem cells are not easy to form a rosette even with a high number [45]. As such, it is evident that the purity of transplanted cells is extremely important to allow differentiation into functional target cells even if ESCs or embryonic RSCs are used.

## Conclusions

Our findings suggested that embryo-derived RSCs exhibited similar properties to those of NSCs, and that transplantation of RSCs had potential to repair the damaged retina. Additionally, intravitreal transplantation of RSCs was a simple feasible delivery way in the treatment of retinal disease. However, many issues about RSCs transplantation still require further extensive investigation, such as the key factors for transplanted RSCs survival, the role of the transplanted RSCs-produced cytokines in the repairment of damaged retina, and the factors that can promote the differentiation of the transplanted RSCs into mature retinal functional cells.

## Data Availability

All the data used to support the findings of this study are included within the article and are available from corresponding author by a reasonable request.

## Abbreviations

AMD: Age-related macular degeneration
RP: Retinitis pigmentosa
PR: Photoreceptors
RPE: Retinal pigment epithelium
PSCs: Pluripotent stem cells
ESCs: Embryonic stem cells
iPSCs: Induced pluripotent stem cells
RSCs: Retinal stem cells
CMZ: Ciliary marginal zone
DMEM: Dulbecco’s modified Eagle’s medium
BrdU: 5-bromo-2’-deoxyuridine
PLL: Poly-L-lysine
H&E: Hematoxylin and eosin
RNL: Retinal neurosensory layer
RPE: Retinal pigment epithelium
CL: Choroid layer
NSCs: Neural stem cells
